# Dr. Charles Bagge Plowright

**Published:** 1910-05-07

**Authors:** 


The death is announced of Dr. Charles Bagge Plowright, at North Wootton, Norfolk, at the age of 61. He took his L.R.C.P. (Edin.) and M.R.C.S. (Eng.) in 1870, graduated M.D. at Durham in 1890, and became an hon. F.R.C.S. in 1893. He was Professor of Comparative Anatomy and Physiology at the Royal College of Surgeons from 1890 to 1894; had practised in King's Lynn and West Norfolk; and for thirty-two years was M.O.H. to Freebridge, Lynn, R.D.C. He was a member of the Lunacy Commission, and had been a local magistrate since 1887. He was a great authority on fungi, his book on this subject being a standard work of reference. His hospital appointments included that of consulting surgeon to the West Norfolk and Lynn Hospital.

